# Optical nano-woodpiles: large-area metallic photonic crystals and metamaterials

**DOI:** 10.1038/srep08313

**Published:** 2015-02-09

**Authors:** Lindsey A. Ibbotson, Angela Demetriadou, Stephen Croxall, Ortwin Hess, Jeremy J. Baumberg

**Affiliations:** 1 NanoPhotonics Centre, Cavendish Laboratory, Department of Physics, JJ Thompson Ave, University of Cambridge, Cambridge, CB3 0HE, UK; 2 Department of Chemistry, Prince Consort Road, Imperial College London, SW7 2AZ, UK; 3 Department of Materials Science and Metallurgy, University of Cambridge, UK; 4 The Blackett Laboratory, Department of Physics, Prince Consort Road, Imperial College, London, SW7 2AZ, UK

## Abstract

Metallic woodpile photonic crystals and metamaterials operating across the visible spectrum are extremely difficult to construct over large areas, because of the intricate three-dimensional nanostructures and sub-50 nm features demanded. Previous routes use electron-beam lithography or direct laser writing but widespread application is restricted by their expense and low throughput. Scalable approaches including soft lithography, colloidal self-assembly, and interference holography, produce structures limited in feature size, material durability, or geometry. By multiply stacking gold nanowire flexible gratings, we demonstrate a scalable high-fidelity approach for fabricating flexible metallic woodpile photonic crystals, with features down to 10 nm produced in bulk and at low cost. Control of stacking sequence, asymmetry, and orientation elicits great control, with visible-wavelength band-gap reflections exceeding 60%, and with strong induced chirality. Such flexible and stretchable architectures can produce metamaterials with refractive index near zero, and are easily tuned across the IR and visible ranges.

Controlling electromagnetic (EM) waves in three dimensions using photonic crystals has stimulated research over several decades. In such structures, the periodic arrangement of dielectric inclusions forbids light propagation inside the sample, opening up energy band gaps in the photonic dispersion. The sub-50 nm-scale structures demanded have so far required electron-beam lithography^1^,^2^, direct laser writing^3^,^4^,^5^,^6^, soft lithography^7^, colloidal self-assembly^8^,^9^, or interference holography^10^. Many promising applications have been identified^11^,^12^, such as filters, optical switches, energy harvesting, ultra-compact low-loss optical waveguides^8^,^13^, very high cavity quality (Q)-factor nanolasers^2^, and manipulation of spontaneous emission^14^,^15^.

The most effective type of three-dimensional (3D) photonic crystal is the woodpile structure^2^,^3^,^4^,^13^,^16^,^17^, in which each layer contains a regular array of rods orientated perpendicular to the rods in the layer below. This architecture has one of the strongest EM interactions, leading to full robust photonic band gaps when constructed with the rods arranged in a face-centred cubic lattice^18^,^19^. However, very few large-scale fabrication techniques can achieve the required nano-features for an optical woodpile, with direct laser writing currently among the most prominent but over prohibitively limited areas. Our methodology here allows for the fabrication of stretchable, bulk woodpile structures composed of metallic wires just a few tens of nanometers wide, which are embedded inside a polymer matrix. Most importantly this allows these structures to be flexible and stretch-tuneable without issues of bowing or collapse^4^,^17^. They also benefit from a high refractive index contrast due to the metal wires which exhibit negative permittivity below the metal's plasma frequency, widening the band gap^20^,^21^.

Gold woodpiles are assembled by stacking large-area polymer films coated in gratings of metal wires, using a simple technique in which the films are captured onto a water surface and then stacked, rolled or folded in customisable orientations to build up an arbitrary number of layers (Fig. 1a–d)^22^. Gratings are first imprinted into a layer of sacrificial polymer, polystyrene sulfonic acid (PSS). Gold is then deposited at glancing angle on top of the PSS grating so that distinct wires are created from shadowing [Fig. 1(a,e,f)], producing nanowires as narrow as 50 nm with thickness 30 nm that span many cm in length.

The gratings of Au wires are then transferred from the sacrificial layer onto polymer films that will be stacked to produce the woodpile. A solution of the desired polymer, typically polystyrene (PS), is spin-cast onto the wire grating so that when the PSS is removed by dissolving in water, the wires are left embedded in the PS. The average thickness of the PS film is calibrated to be half the pitch of the grating by altering the spin speed and concentration of the polymer solution, so that once stacked the vertical periodicity (Λ_z_) of the woodpile (equivalent to the thickness of two grating films due to their perpendicular orientations) equals the lateral periodicity (Λ_x,y_) of the gratings. At this stage the free-standing films on water produced can be accessed from above or below and collected on top of other films in any orientation. A bilayer of two perpendicular grating films that forms the basis for a woodpile structure can be easily assembled by collecting one film on top of another with a relative angle of 90° [Fig. 1(b)]. Once dry, this bilayer can be similarly released [Fig. 1(c)] and rolled up or stacked onto further films to build up many woodpile layers [Fig. 1(d)].

Scanning electron microscope (SEM) images [Fig. 1(e,f)] show the uniformity of the wire gratings after transfer onto polymer films (torn in (f) to evidence the robustness of the surrounding wires). The stacked layers in the woodpile are very uniform, as seen in cross-sections produced by focussed-ion beam (FIB) milling of a stack of 8 bilayers cut at 45° to both wire grating directions [Fig. 1(g)]. By FIB-sectioning successive slices a three-dimensional image of the full structure is built up (Supplementary Video 1). Our assembly technique aims to take advantage of the locking of grooves on successive bilayers in step Fig 1(d). It is currently not clear how precise the alignment is, which comes from interpreting Fig 1(g) and is inconclusive. Disorder in lateral alignment is expected to reduce the bandgap reflection, but because the EM interaction is most strongly dependent on the orientation of the wires in successive layers, which is controlled to within a few degrees, this effect should be small. Each individual wire layer is highly flexible [Fig. 1(e)] and large area [Fig. 1(h)]. Woodpiles from wire grating films of pitch Λ = 556 nm rolled onto a square glass rod appear brightly coloured due to diffraction [Fig. 1(i)]. Regions of different colour along each rod face indicate varying numbers of woodpile layers while different faces of the rod show different colours due to varying diffraction angles. Spectra are uniform in regions between imperfections (with areas around 10^4^ μm^2^) and between these regions across the entire sample, with local defects currently affecting over 10% of the total stack area (but defects in <1% of each individual layer). Imperfections are sparsely present in each of the 16 stacked films for an 8-bilayer stack, caused by film wrinkling, dirt, and imprint defects. These would be greatly reduced for an automated process in a clean room environment.

We investigate the optical properties of a range of gold wire woodpiles initially with periodicity Λ = 278 nm. Gratings of this pitch have minimal diffraction at visible wavelengths and the first order photonic band gap is expected around 900 nm (λ_g_ = 2n_eff_Λ) with the second order gap around 450 nm. The linearly polarised incident light has electric field (E) either perpendicular or parallel to the uppermost grating wires in the stack. The optical reflection spectra (Fig. 2a) exhibit first and second order photonic band gaps in both polarisations (red and blue lines) at the expected wavelengths, highlighted in yellow and confirmed by numerical Finite Integration Techniques (FIT, dashed lines). The polarisation anisotropy observed from the stack in both experiment and theory arises from the different TE/TM excitation of the last wire, which interacts directly with the adjacent substrate.

The flexibility of this fabrication technique allows for creating continuous Au coatings (by sputtering uniformly) or omitting the Au completely (Fig. 2b,c), which in both cases eradicates the band gap around 900 nm. This can be explained from polarization anisotropy in the woodpile. When the electric field oscillates along the wires electrons travel freely, however when the field oscillates transverse to wires, the confined electrons resonate as in a metallic nanoparticle. Polarized light thus interacts very differently with successive layers possessing perpendicular wire orientations (Supplementary Fig. S3). This effect disappears for gratings with absent or continuous gold coatings, removing the polarization anisotropy, and thus halving the vertical periodicity of the stacks. As a result, the first order band gaps are at half their previous wavelength (450 nm) as confirmed by FIT simulations (dashed). An equivalent dependence of band gap position on vertical periodicity Λ_z_ is seen in multilayer planar stacks with the original and halved periodicities exhibiting first order band gaps at 850 nm and 450 nm respectively (Supplementary Fig. S7).

For the air woodpile without gold, strong refractive index contrast provided by air trapped between the PS layers (black holes in Fig. 1g) gives peak reflectivities >80% (Fig. 2c). Such flexible Bragg reflectors made only from polymer and air are very difficult to achieve by other means and have a much larger refractive index contrast than stacks built from two different polymers. This yields spectrally-broad and high reflectivity flexible mirrors, useful for diverse applications.

The woodpile band gaps arise from 3D periodicity, as seen in the dispersion relations of the infinite structure and their field distributions (Supplementary Figs. S4, S8). The measured band gap (Fig. 2a) is found to be 60% broader than from planar stacks, showing the benefit of coupling to plasmonic effects in full 3D rather than just 1D planar periodicity. These metamaterials have refractive index near zero (Supplementary Fig. S5), that is highly promising to enhance nonlinear optical switching which dominates wherever the linear index is negligible.

The behaviour of these woodpile structures changes when moving from the photonic crystal regime to the metamaterial regime, requiring now smaller periodicities that satisfy the condition λ > 2nΛ_x,y,z_ at optical wavelengths. Woodpiles are easily fabricated using gratings of period 139 nm and stacking films of thickness 70 nm. The band structure of this 3D optical metamaterial shows mixed behaviour, with photonic-crystal-like bands coupled to flat isotropic plasmonic bands (Supplementary Figs. S8b). The reflectivity of a woodpile with Λ_z_ = Λ_x,y_ = 139 nm [Fig. 3(a)] shows a peak at around 700 nm, completely shifted from the expected linear scaling with periodicity (which predicts the bandgap at 430 nm). This effect is confirmed by FIT simulations (dashed lines), which also show dramatic changes in effective refractive index (Supp. Figs. S5, 6). For λ > 1200 nm the reflectivity increases, indicating the new red-shifted effective plasma frequency for this metallic wire mesh^23^,^24^,^25^, also confirmed by simulations.

The construction flexibility allows us to explore anisotropic woodpiles, for instance with unequal vertical and lateral periodicities. Gold wire woodpiles are fabricated with thicker films giving Λ_z_ = 278 nm but using gratings of half the pitch, Λ_x,y_ = 139 nm. While the band gaps in this anisotropic grating woodpile [Fig. 3(b)] are at similar wavelengths to the cubic woodpile [Fig. 2(a)], the maximum reflectivity is almost doubled. This effect matches simulations, and originates from hybridization of the metamaterial band gap arising when propagating modes reach the Brillouin edge, with the first vertical Bragg resonances between neighbouring wire layers. We note that the strong band gaps for this anisotropic woodpile with 60% reflectivity exceed comparable woodpile photonic crystals in the literature but now at much shorter wavelengths than ever before^1^,^8^,^10^,^17^. Optical woodpile metamaterials can thus be successfully accessed through wire gratings, in which the optical thickness and scattering strength can be independently tuned.

This stacked woodpile route to 3D metamaterials is promising for its flexibility and scalability. In contrast to gyroid systems^26^,^27^ in which the architecture is spontaneously formed from block co-polymer phase separation, here the structure is single domain and can be selectively oriented in any fashion, but is also large area unlike recent lithographic approaches using severed wires^31^. For instance chiral metamaterials can be produced by stacking with a progressive rotation in the bilayers (Fig. 3c), or by rolling around a cone-shaped rod. With only 7 chiral periods (28 individual layers), these woodpiles produce large circular dichroic reflectivity response around the bandgap, *σ* = (*R*_+_ − *R*_−_)/(*R*_+_ + *R*_−_) > 25% (with *R*_±_ the reflectivity of each circular component), as well as extremely strong circular polarization conversion (absent in normal metamaterials, Supp. Figs. S9, S10). Simulations (dashed) show that circular dichroism exceeding 70% is expected, with perfect alignment and no air-gaps. Furthermore we find both twisted woodpile photonic crystals and metamaterials can show this strong chiral response^32^. Further optimization of the optical properties can be easily achieved by tuning the grating pitch and cross-section of the wires. Our approach is a significant advance for scalable nanofabrication of optical 3D photonic crystals and metamaterials that will find applications in the coating of large arbitrarily-curved surfaces to provide optical filters and switches, sensors, cloaking, light harvesting, and enhanced photochemical devices. In addition, the artificially obtained and tunable low refractive indices in the metamaterials regime give great promise for diverse 3D nonlinear optical devices.

## Methods

### Sample Fabrication

A wide range of master gratings can be utilised, and here Si gratings of period ≥139 nm and aspect ratios of 0.4 are replicated into the fluoropolymer ethylene-tetrafluoroethylene (ETFE)^28^ as discussed elsewhere^29^. The size of a resulting photonic crystal is limited only by the area of the master grating. For the wires, sputtering is used rather than evaporation as radiation during the electron-beam evaporation otherwise cures the PSS film, making it difficult to remove later. The sacrificial material PSS is chosen for its high solubility in water and is easily removed from beneath the PS grating film by gradually immersing the sample into a water bath. This allows the hydrophobic PS grating film to be captured onto the water meniscus as it is released, where it is kept completely flat by the high surface tension. Accurate angular alignment between stacked bilayers can be achieved after the film is collected since a layer of water is temporarily trapped between the films, allowing them to move across each other. As the water evaporates the top film conforms to the bottom film giving large uniform regions and minimizing wrinkles.

### Analysis

For cross sections, a dual-beam FIB-SEM microscope is used (FEI Helios Nanolab 600) and samples are prepared by cleaving, mounting on aluminium SEM stubs, then partially coating with platinum for protection during milling. Trenches are milled around the region of interest with a gallium ion beam, from which successive cross-sections are milled and imaged to provide the 3D structure. The alignment of wires in next-nearest-neighbour layers appears to be randomly shifted despite potential for locked alignment through their surface textures.

### Experimental

Reflection spectroscopy: Reflection spectra at normal incidence are measured over λ = 400–1600 nm using a modified microscope with confocally-arranged collection fibres taking light to visible and near-infrared spectrometers.

### Simulations

The numerically calculated reflection spectra were obtained using a commercial code based on Finite Integration Techniques (CST GmbH, Darmstad, Germany). The simulated reflection accounts for scattering and absorption from granularities in the gold wires by using a complex permittivity with imaginary part increased by 50% due to the decreased conductivity of the Au (as previously studied in Au gyroid metamaterials^30^), which reduces the peak reflection (Supplementary Figs. S1 and S2). Full convergence is confirmed at both long and short wavelengths. The band structures of the various woodpiles were calculated numerically using a Finite Difference Time Domain (FDTD) commercial code (Lumerical Solutions). The same adapted dielectric permittivity of gold is used to model the metal structures of the woodpile, and again convergence is ensured. A set of 20 dipoles sources are distributed through the unit cell, capable of exciting all modes. Forty distributed time monitors collect the fields over time. Most fields decay quickly due to destructive interference, except fields due to modes of the structure, which continue to propagate indefinitely. The frequencies of these modes are detected from a Fast Fourier Transformation of the fields in time.

## Author Contributions

Experiments were planned and executed by J.J.B. and L.I.A. The data were analyzed by L.I.A. and J.J.B., FIB-SEM sectioning was performed by S.C., the simulations and theory were made by A.D. and O.H., and all authors contributed to the manuscript.

## Supplementary Material

Supplementary Information Supplementary Information

## Figures and Tables

**Figure 1:**
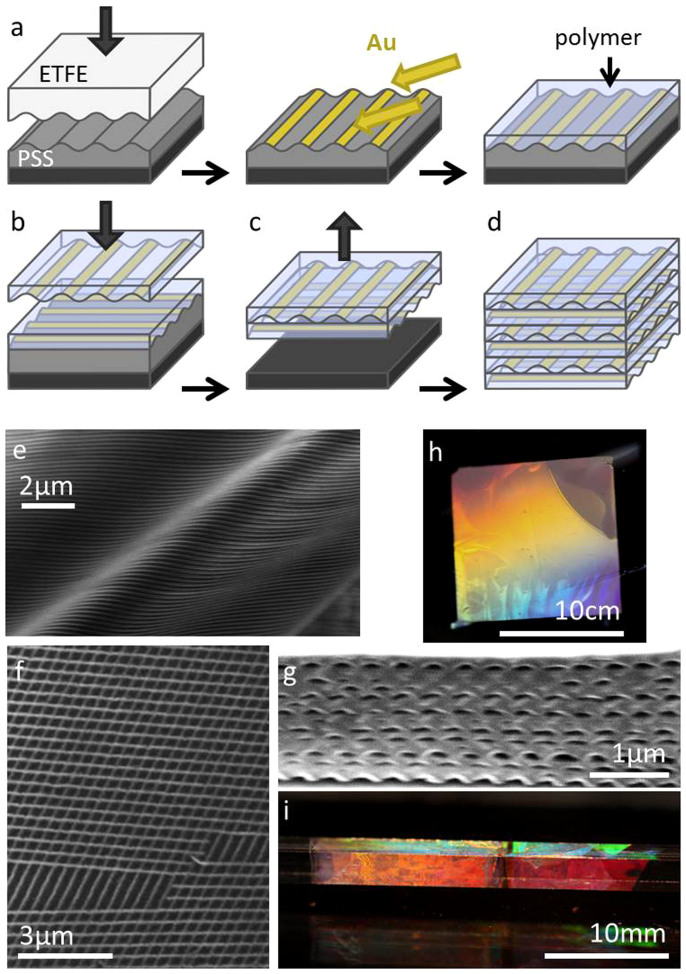
Fabrication of Au wire woodpiles. (a), PSS film imprinted with ETFE grating, shadow deposition of Au wires, and polymer film spin-cast over wires. (b), Wire grating film released and collected onto another grating film at 90°, (c), bilayer grating film released and stacked/rolled to form (d), multilayer woodpile. (e),(f),(g), SEM images of (e), flexible single wire layer, (f), bilayer Au wire grating film (angularly misaligned here) and (g), FIB-milled cross-section of woodpile with 8 bilayers. (h),(i), Optical white light images of (h), single layer, and (i), woodpile rolled onto a glass rod, grating period 556 nm.

**Figure 2:**
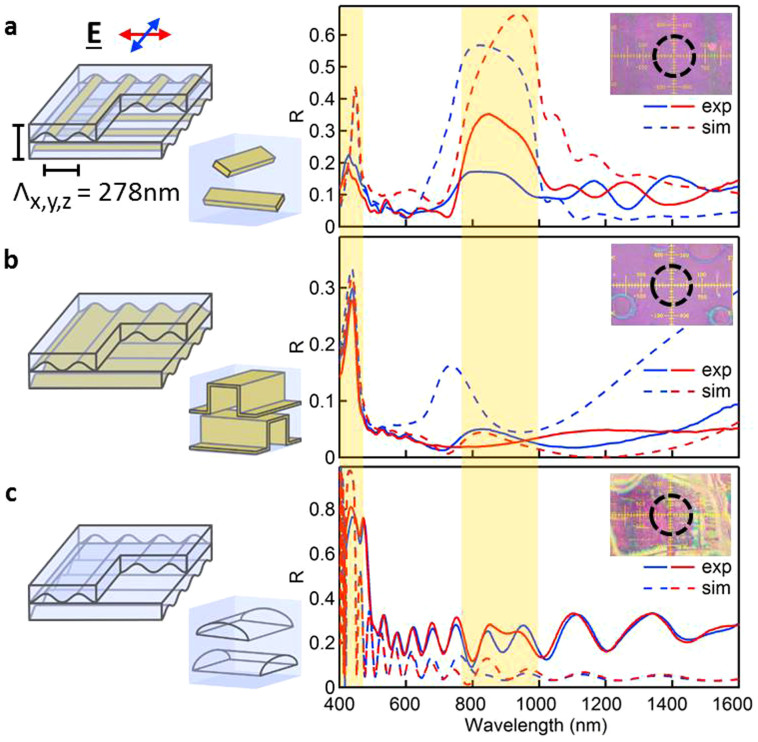
Reflectivity of different stacks with Λ_z_ = Λ_x,y_ = 278 nm, built from 8 bilayers (unit cells illustrated). (a) Woodpile of Au wires in PS, (b) stack of PS grating films each uniformly coated with 6 nm Au, (c) stack of PS grating films with no Au. Linearly polarised optical spectra (and simulations) shown by solid (dashed) lines, with E field across (along) the wires of the top layer in red (blue). Insets: Reflection images, dashed circle outlines 50 μm collection spot. Yellow shading shows expected bandgaps.

**Figure 3:**
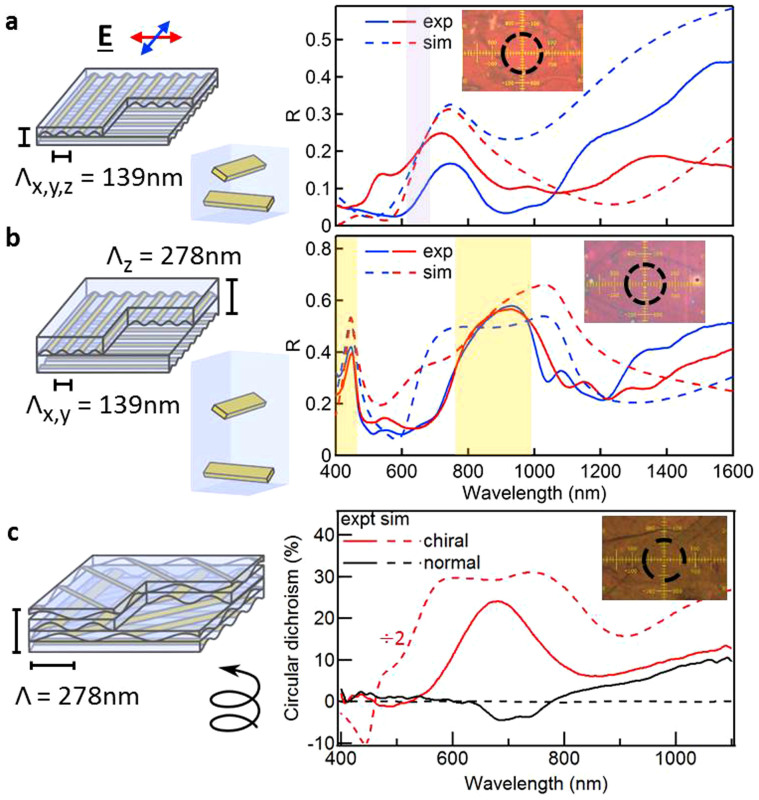
Reflectivity of anisotropic and chiral Au wire woodpiles. (a),(b), Stacks with Λ_x,y_ = 139 nm and (a), Λ_Z_ = 139 nm (9 bilayers) and (b), Λ_Z_ = 278 nm (8 bilayers). Linearly polarised optical spectra (simulations) shown by solid (dashed) lines, with E field across (along) the wires of the top layer in red (blue). Insets: Reflection images, dashed circle outlines 50 μm collection spot. Yellow shading shows same photonic band gaps as Fig. 2. (c), Stacks with 45° rotation between wire layers, giving chiral reflection response shown (dashed is simulations 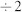), for both normal and chiral woodpiles.
