# Supplementary material for: Optical nano-woodpiles: large-area metallic photonic crystals and metamaterials
Source: Sci Rep. 2015 Feb 9;5:8313. doi: 10.1038/srep08313 (PMC4321162; doi:10.1038/srep08313)
Supplement: Supplementary Information [file srep08313-s1.pdf]

# Optical nano-woodpiles: large-area metallic photonic crystals and metamaterials

Lindsey A. Ibbotson, Angela Demetriadou, Stephan Croxall, Ortwin Hess*, Jeremy J. Baumberg*

## Contents

**Supplementary Figures**
**Figure S1.** Effect of air gaps in Λ= 278nm gold wire woodpile stacks
**Figure S2.** Effect of air gaps in Λ= 139nm gold wire woodpile stacks
**Figure S3.** Absorption/scattering of single Au wire gratings on PS films with glass substrates
**Figure S4.** Simulated electric field distributions in unit cells of infinite woodpile stacks
**Figure S5.** Extracted refractive index, permittivity, and permeability of Λ= 278nm gold woodpile structure
**Figure S6.** Extracted refractive index, permittivity, and permeability of Λ= 139nm gold woodpile structure
**Figure S7.** Modelled reflectivity from multilayer stacks of flat PS and Au
**Figure S8.** Band diagrams for infinite woodpile stacks
**Figure S9.** Chiral reflectivity
**Figure S10.** Chiral polarisation conversion

**Supplementary Videos**
**Video 1.** Successive cross-sections through an 8-bilayer woodpile stack

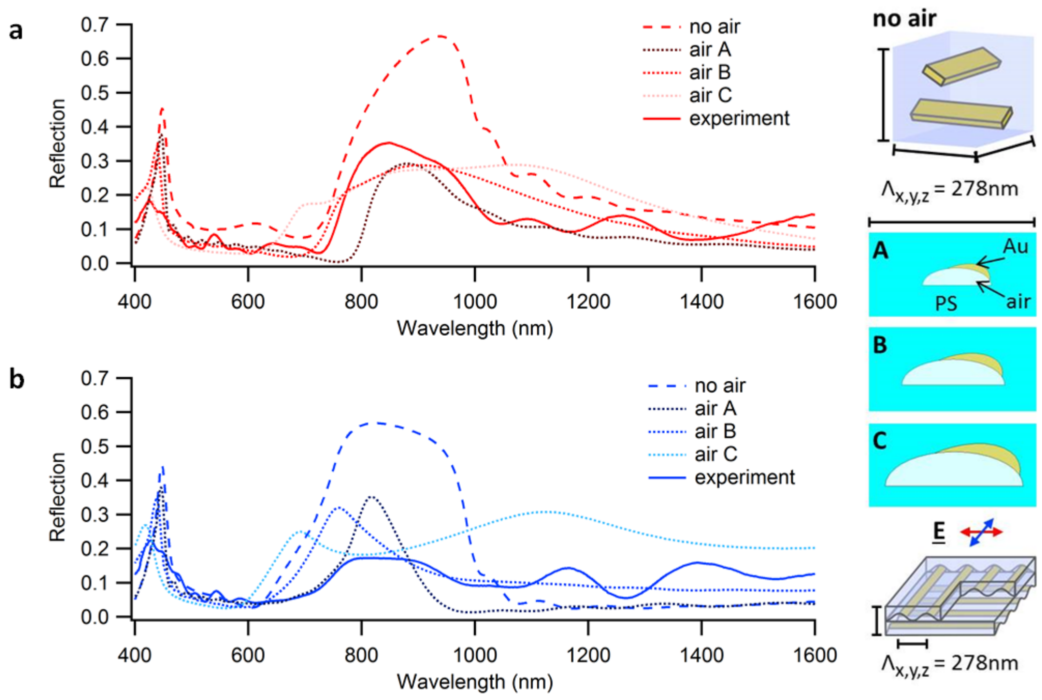


**Supplementary Figure S1: Effect of air gaps in Λ= 278nm gold wire woodpile stacks.** Simulated (experimental) reflection of 10 (8) bilayers with/without air gaps of varying size shown by dotted/dashed (solid) lines, electric field polarised across (along) uppermost wires shown by red (blue) lines. Insets show the varying cross-sections of wires and air gaps used in model (labelled A,B,C). Simulations with increasing fill fractions of air and gold decrease reflection and shift and weaken the photonic band gap. Small air pockets are present in fabricated woodpile stacks due to grating profiles on the bottom of stacked films, accounting for the reduced reflectivity.

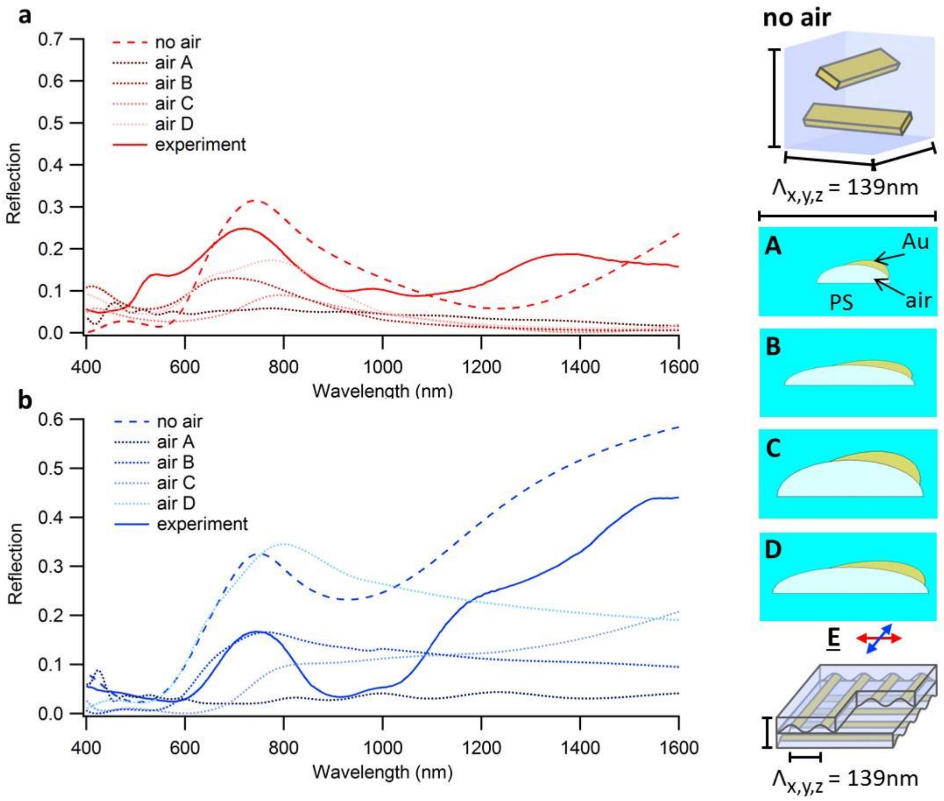


**Supplementary Figure S2: Effect of air gaps in Λ= 139nm gold wire woodpile stacks.** Simulated (experimental) reflection of 10 (8) bilayers with/without air gaps of varying size shown by dotted/dashed (solid) lines, electric field polarised across (along) uppermost wires shown by red (blue) lines. Insets show the varying cross-sections of wires and air gaps used in model. Simulations with increasing fill fractions of air and gold decrease reflection and shift and weaken the photonic band gap peak. The shape of the peak shows a sensitive dependence on the shapes of wires and air gaps as their aspect ratio is varied.

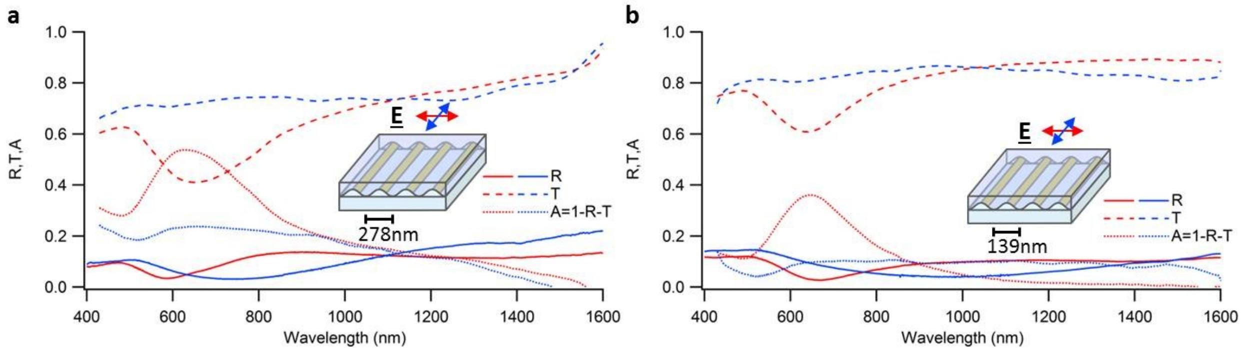


**Supplementary Figure S3: Absorption/scattering of single Au wire gratings on PS films with glass substrates.**
Normal incidence reflection (R, solid lines) and transmission (T, dashed lines) shown with grating pitches (a) 278nm and (b) 139nm, film thickness equal to half the pitch. Absorption/scattering (A, dotted lines) is calculated using R+T+A=1. Spectra from incident light linearly polarised with its electric field across (red lines) wires of both pitches show a particle plasmon peak in absorption at 600-700nm that is not present in the orthogonal polarisation (blue lines)[1,2].

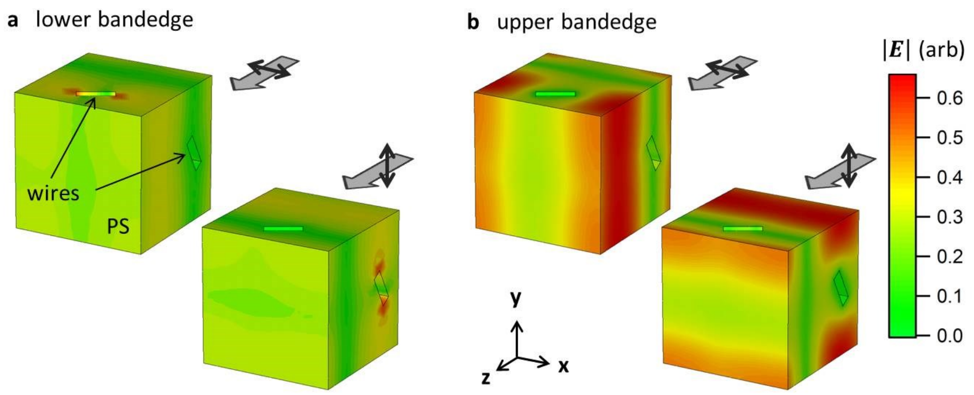


**Supplementary Figure S4: Simulated electric field distributions in unit cells of infinite woodpile stacks** from modes at $\boldsymbol{k} = (0,0,\pi/a)$ and energies at the (a) lower and (b) upper 1st order band edge, for polarisations as shown. Field distributions simulated in woodpiles of perfect electric conductor (PEC) for spectral positions either side of the first order photonic band gap show typical 3D photonic crystal behaviour, with the electric field localised at high index (wire) regions for the lower band edge [Fig.S4(a)] and at low index (between wire) regions for the upper band edge [Fig.S4(b)]. The degenerate polarization modes at each energy show periodicity in the field in both the vertical and lateral directions, confirming 3D metamaterial behaviour for these woodpiles.

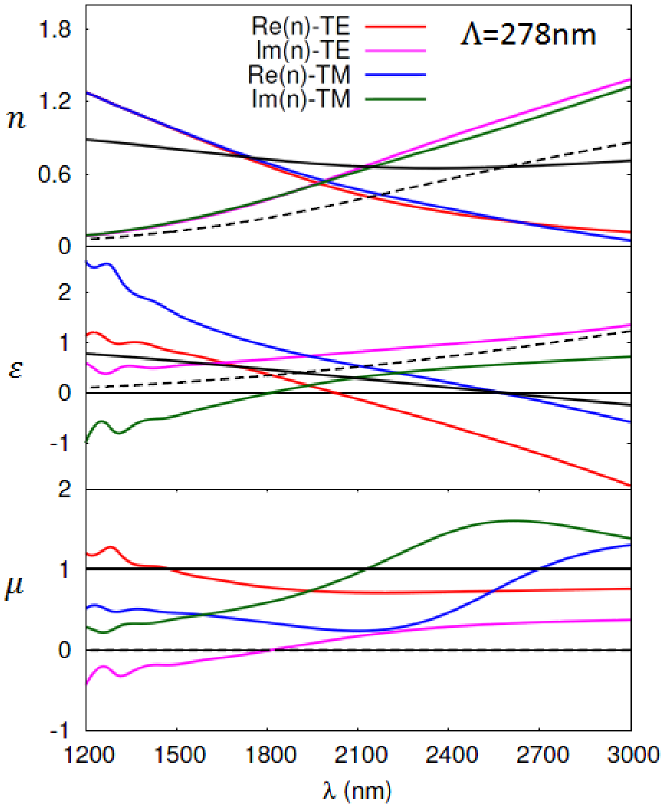


**Supplementary Figure S5: Extracted refractive index, permittivity, and permeability of gold woodpile structure**, extracted from the S-parameter simulations of reflection and transmission, for the 8-bilayer samples with $\Lambda_z = \Lambda_{x,y} =$ 278nm. Polarization is TM (field across top wires) and TE (field along top wires). In this retrieval the presence of the substrate was taken into account. Note that the effective parameters are plotted for wavelengths smaller than 1000nm, where the structure behaves as a metamaterial (i.e. an effective medium). The black lines use an effective dielectric permittivity model[3,4] for the gyroid and assume a magnetic permeability of 1 (solid = Re, dashed = Im).

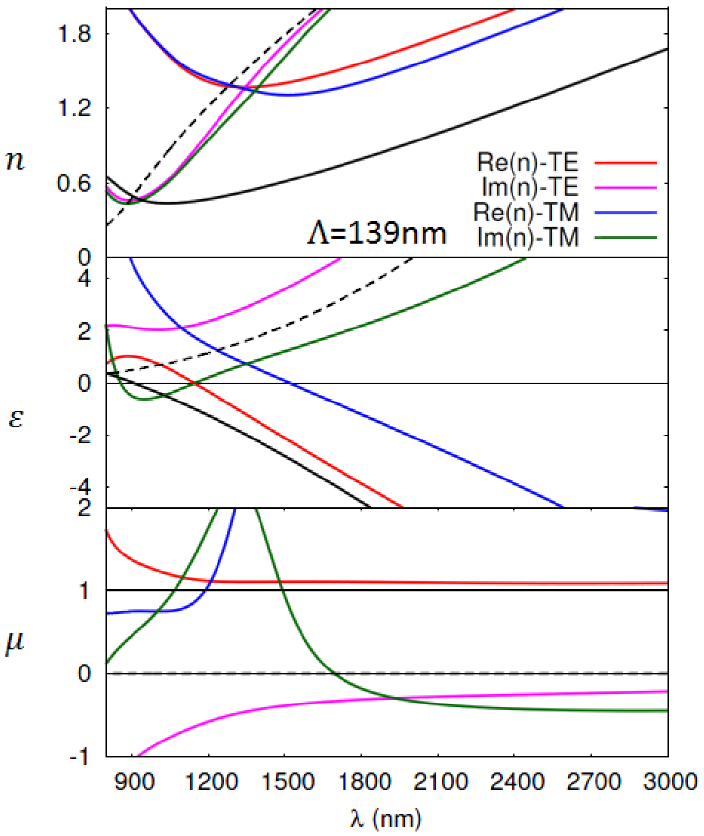


**Supplementary Figure S6: Extracted refractive index, permittivity, and permeability of gold woodpile structure**, extracted from the S-parameter simulations of reflection and transmission, for the 8-bilayer samples with $\Lambda_z = \Lambda_{x,y} =$ 139nm. Polarization is TM (field across top wires) and TE (field along top wires). In this retrieval the presence of the substrate was taken into account. Note that the effective parameters are plotted for wavelengths smaller than 700nm, where the structure still behaves as a metamaterial (i.e. an effective medium). The black lines use an effective dielectric permittivity model[3,4] for the gyroid and assume a magnetic permeability of 1 (solid = Re, dashed = Im).

In **Supplementary Figures S5,S6** a retrieval method was used to extract the refractive index for the graphs above. To account for the presence of the substrate attached on the woodpile metamaterial, the retrieved electromagnetic parameters for the structure is derived, improving existing methods[5,6]. The effective impedance ($Z_{\mathrm{eff}}$) and refractive index ($n_{\mathrm{eff}}$) of the metamaterial are given by:

$$Z_{\mathrm{eff}} = \pm Z_1 Z_3 \sqrt{\frac{(1+R)-T}{Z_3^2(1+R)^2 - Z_1^2 T^2}}$$

$$n_{\mathrm{eff}} = \frac{\ln\left(X \pm \sqrt{1-X^2}\right)}{ikd} + \frac{2m\pi}{kd}$$

where $X = \frac{Z_1 T^2 + Z_3(1-R)^2}{T(Z_1+Z_3+(Z_1-Z_3)R)}$, $m$ is an integer, $d$ is the thickness of the metamaterial sample, $R$ and $T$ the reflection and transmission in complex format, $Z_1$ and $Z_3$ are the impedances of the two media enclosing the metamaterial slab. The choice of branch for $Re(n_{\mathrm{eff}})$ and the choice in signs are determined mainly by causality, which enforces that $Re(Z) > 0$ and $Im(n) > 0$. The effective electric permittivity and magnetic permeability of the metamaterial are obtained through $\varepsilon_{\mathrm{eff}} = n_{\mathrm{eff}}/Z_{\mathrm{eff}}$ and $\mu_{\mathrm{eff}} = n_{\mathrm{eff}} Z_{\mathrm{eff}}$. Since the reflection and transmission for the two polarizations are different, the effective impedance retrieved from each polarization is different. This is due to the fact that the last wire of the woodpile, which is excited differently from each polarization, touches the substrate, changing the reflection from this interface and hence affecting the retrieved value of the impedance. The effective refractive indices from the two polarizations are however very similar.

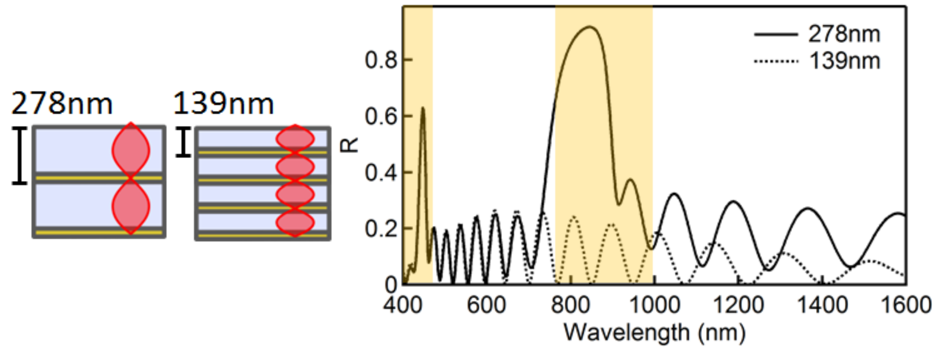


**Supplementary Figure S7: Modelled reflectivity from multilayer stacks of flat PS and Au**
Modelled reflectivity using transfer matrix simulations from stacks of flat PS and 5nm Au, of same total thickness and period as Fig.2(a-c) (solid line), and with half all vertical dimensions and twice the number of layers (dashed line), see schematic. Yellow shading shows 1st and 2nd order photonic band gaps

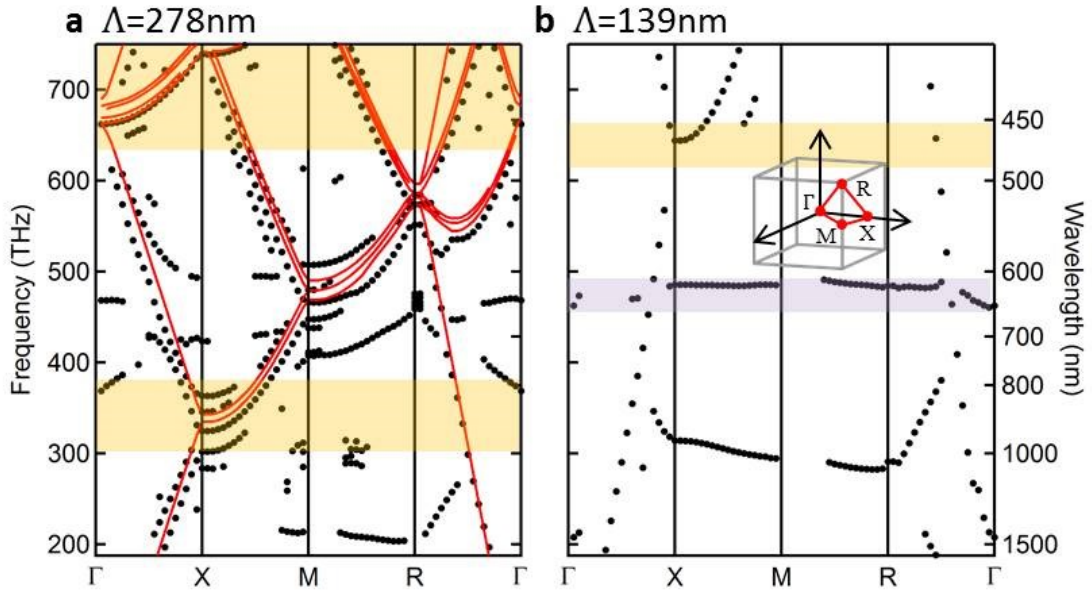


**Supplementary Figure S8 | Band diagrams for infinite woodpile stacks**. (a) Au wires (black dots) and air gaps (red lines) in PS with $\Lambda_z = \Lambda_{x,y} =$ 278nm (as Fig.2a,c). (b) Au wires with $\Lambda_z = \Lambda_{x,y} =$ 139nm (as Fig.4a). Yellow (purple) shading shows 1st and 2nd order photonic (plasmonic) band gaps.

In **Supplementary Figures S8**, the band diagrams are calculated using a Finite-Difference Time-Domain (FDTD) code. While the air-only structure shows a simple $k_z$ periodicity (red lines), the Au wire woodpile has wider band gaps arising from the full 3D periodicity (black dots) as well as localised plasmonic resonances (ie. dispersion-less bands). Thus, coupling to plasmonic effects enhances the photonic-crystal bandgap widths (Supplementary Fig. S3).

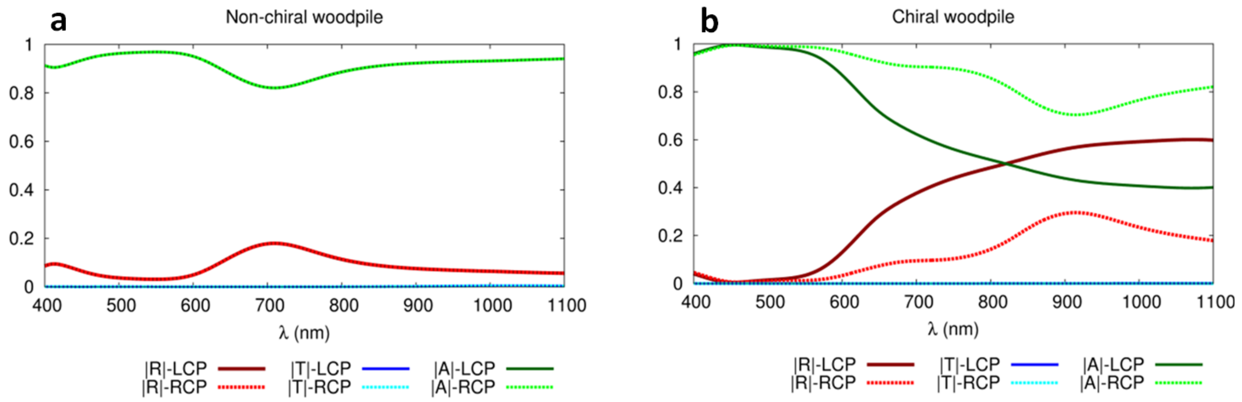


**Supplementary Figure S9 | Chiral reflectivity.** Simulated Reflectivity (R), Transmission (T), and Absorption (A) of the right- (RCP) and left- (LCP) circular polarisations, for a thick woodpile structure which is (a) non-chiral, and (b) chiral (as in Fig3c).

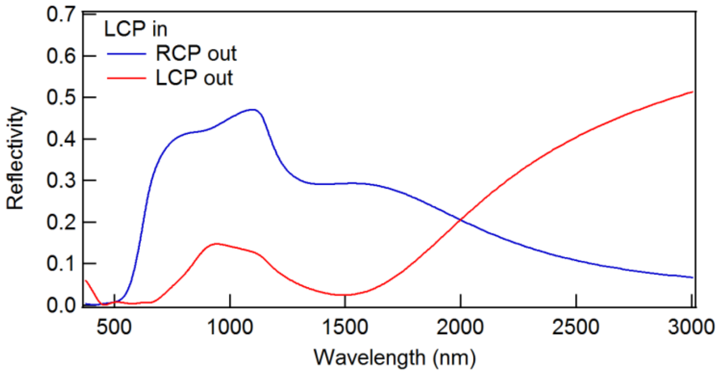


**Supplementary Figure S10 | Chiral polarisation conversion.** Simulated reflectivity from chiral multilayers (as from Fig3c), for left-circular polarisation input on a right-twist chiral metamaterial, showing strong polarisation conversion to right circular polarised light.

**Supplementary Video 1: Successive cross-sections through an 8-bilayer woodpile stack** on silicon milled vertically using FIB at 45° to both wire directions, slices of 20nm removed in each frame. The three-dimensional structure built up from these sections shows the wires are not perfectly aligned in the vertical direction. The SEM contrast shows black holes indicating air pockets, and bright spots indicating gold wires. The imaged sample is a preliminary version and has thinner films and less gold than the final samples whose optical data are presented in this paper.

**Additional references:**

1. Ahn, S-W. *et al.* Fabrication of a 50 nm half-pitch wire grid polarizer using nanoimprint lithography. *Nanotechnology* **16,** 1874-1877 (2005).
2. Schider, G. *et al.* Optical properties of Ag and Au nanowire gratings. *J. Appl. Phys.* **90,** 3825-3830 (2001).
3. Belov P.A. *et.al.* Strong spatial dispersion in wire media in the very large wavelength limit, *Physical Review B* **69**, 113103, (2003).
4. Demetriadou A. *et.al.* Taming spatial dispersion in wire metamaterials, J. of Physics: Condensed Matter **20**, 295222, (2008).
5. Chen X. et.al. Robust method to retrieve the constitutive effective parameters of metamaterials, *Physical Review E* **70**,016608, (2004).
6. Smith D. *et.al.* Electromagnetic parameter retrieval from inhomogeneous metamaterials, *Physical Review E* **71**, 036617, (2005).
